# Being the Facilitator: A Brief Research Report on the Motivation of the Choreographer and Dance Maker to Work With Heterogeneous Groups in a Community Dance Setting

**DOI:** 10.3389/fpsyg.2021.601033

**Published:** 2021-02-22

**Authors:** Mia Sophia Bilitza

**Affiliations:** Rehabilitation Science in Music and Movement in Rehabilitation and Pedagogy in Disability, TU Dortmund University, Dortmund, Germany

**Keywords:** motivation, inclusion, dance facilitator, art project, community dance, inclusive dance

## Abstract

In inclusive dance settings, where people with different abilities and talents come together, the role of facilitators is essential in guiding the process of inclusion. Their behavior gives sensitive information to the individual about one’s status within the own-group affiliation (De Cremer, 2002, p. 1336). Even today, very little research on the motivation for facilitating inclusivity in dance contexts exists. This case study will examine the facilitator’s motivation by juxtaposing current theory next to experiences of seven experts of contemporary dance facilitation in Europe. Good opportunities for meaningful interactions can be created in a dance setting: it promotes a deeper sense of community, gives us the feeling of belonging, generates respect and inclusion, and helps to prevent the feeling of loneliness (Elin and Boswell, 2004; Kaufmann, 2006; Whatley, 2007). This research report sheds light on the motivation of being the facilitator of dance for heterogeneous groups and reveals three factors from the data. First, to be led by an artistic motivation, second, to have a vision in terms of changing the society, and third, to have another personal motivation. The motivation of the facilitator is regarded as highly important for inclusive work, as the person who facilitates plays a key role in these successful processes of inclusion (Miesera et al., 2019).

## Introduction

Our communities are diverse: People of different age, gender, education, social heritage, disability, religious beliefs, and sexual orientation are living together. Participation must be achieved, regardless of abilities and background (United Nations, 2006)–also in the arts. This is why, in the sense of an educational political discourse, there is a necessity to identify behaviors, structures, or methods, which address the challenges of inclusive cultural education.

Community dance can be seen as a means to achieve inclusion, as opportunities for meaningful interactions can be created in a dance setting: it promotes a deeper sense of community, gives us the feeling of belonging, generates respect and inclusion, and helps to prevent the feeling of loneliness (Elin and Boswell, 2004; Kaufmann, 2006; Whatley, 2007). Amans (2017, p. 4) suggests that community dance, as a non-elitist form of dance, creates opportunities for anyone, because it disregards any differences. It brings people together by using movement, especially inspiring or motivating people who usually do not–or for different reasons anticipate that they cannot–dance.

The qualities of community dance artists are defined in the United Kingdom by the organization “People Dancing” and their established guidelines “National Occupational Standards for Dance Leadership” (NOS). NOS offers a set of agreed and formally recognized standards of what a professional “needs to do, know and understand in order to carry out a particular job, role or function.” (People Dancing the foundation for community dance, 2011). Also outside the United Kingdom, several guidelines have been developed in order to guarantee qualified (dance) facilitation (cf. Bäcker et al., 2009; Seidel et al., 2009; Quinten et al., 2020). Nevertheless, in all these dance specific standards, little information can be found on the relevant facilitators’ motivation to work with heterogeneous groups.

In order to inspire more professionals to work inclusively with all people regardless of (dis)ability, race, class, or gender in the field of dance, it is important to ask: What motivates contemporary dance artists to work inclusively and to get involved in the community?

This research report takes a look into the subject of analysis of community dance facilitation. The paper offers information from data collected from a sample of expert interviews in the professional field. The research report explores these selected experts’ motivation, while at the same time highlighting the great need for constant further evaluation.

From the psychological view, Heckhausen and Heckhausen (2018) suggest that motivation is always a product of the person in combination with the given situation: “(a)n individual’s motivation to aspire to a certain goal is influenced by person factors and by situation factors, including the anticipated outcomes of actions and their consequences” (2018, p. 4). Also, Atkinson (1964) agrees that motivation is correlated to several individual factors, as motivation always stands in relation to the analysis of the diversity of factors which lead, direct, or decide on a person’s activity. “The study of motivation has to do with analysis of the various factors which incite and direct an individual’s actions” (1964, p. 1) This shows that there are several reasons, such as the expected outcomes or consequences of a project and personal factors, which could all decide on the motivation to engage in dance facilitation. It is therefore interesting to research further on exactly which factors lead to the motivation of being the dance facilitator of heterogeneous groups in the community.

There is evidence from a more general inclusive educational perspective that a correlation can be found between the facilitator’s personal attitudes and concerns about inclusion and their effect on the intention to teach inclusively (Miesera et al., 2019). In their research, Miesera et al. (2019) found out that “apart from the knowledge and skills in teaching inclusive classes, teachers’ attitudes, self-efficacy, and concerns (.) strongly affect the implementation of inclusion in the classroom” (Miesera et al., 2019, p. 103).

Keeping this in mind, it seems even more important to ask for the facilitator’s motivation, which in turn leads to the action of initiating dance projects with highly heterogeneous groups. ArtWorks Cymru, the Welsh partnership program of People Dancing, investigated intentions in community dance settings. Their results suggest the projects and the facilitators’ most important intentions to be:

(a) *Artistic and professional*, meaning that facilitators should have and continue a constant further development in their own training and artistic expertise.
(b) *Relevant and inclusive*, meaning that practices should be revised by their relevance for the participants and accessible for non-professionals to engage in.
(c) *Inspiring, challenging, and engaging*, referring to the facilitators’ own practice which they can bring into the practice and share with the participants. Also, the key objectives of the program should be transmitted to all active parties of each project (ArtWorks Cymru, 2015).

Looking at the motivation to teach inclusively–and having these standards in mind–it could be assumed that there is quite a high level of motivation needed in order to fulfill all these high standards. Whatley and Marsh (2018) agree that working in the field of dance, while making inclusion possible, is just as highly challenging as fruitful: “differently abled dancers show how no bodies are static” (Whatley and Marsh, 2018, p. 4), their diverse abilities create spaces for unforeseen creative opportunities and an environment in which everybody is able to be seen equally, as unique and to be valued positively in their differences (Whatley, 2007). Having these positive intentions of inclusive dance work in mind, it is important to ask to what extent the motivation plays a role in successful dance facilitation. This is why there is a need to look at the facilitator’s motivation in detail.

With time, community dance has–over the past 30 years in the United Kingdom and about the past 20 years in Germany–openly taken the challenge of making dance accessible for a wide range of people within their communities (Bartlett, 2017). Experiences and impacts on the lives of people who have participated in a community dance project have been made scientifically evident. Evidence shows that especially an inclusive approach in dance projects addresses all learners, it furthers social, cognitive, motoric, as well as emotional skills and it supports the feeling of belonging, and thus peaceful interaction (cf. Gardner et al., 2008; Bäcker et al., 2009; Skoning, 2010; Facci, 2011; Quinten, 2016; Burridge and Nielsen, 2018).

This study maintains a focus on the motivation of the facilitators, as they are seen to be the ones initiating these positive impacts on their groups. In fact, their fair behavior gives sensitive information to the individuals about each participant’s own status of group affiliation (De Cremer, 2002). This research report sheds light on working with heterogeneous groups and lays open the motivation of seven artists from the field.

## Methods

This study deals with the hypothesis that the motivation of dance facilitators plays an important key role in the success of dance projects with heterogeneous groups. Data are taken from expert interviews with prestigious freelance male and female dance facilitators who mainly work in or for the community and are in the network of the researcher^1^. The experts were chosen based on their outstanding expertise, which includes a professional dance education as well as at least 10 years of professional working experience. Some of them have been working over 30 years internationally as community dance facilitators. Due to research economic reasons, the number was limited to seven interviews. All artists voluntarily gave written consent for their data to be used as material for scientific research and for their name to be mentioned.

The interviews have been held as dyads, face to face, and over Skype with: Dana Caspersen (conflict specialist, dancer, and choreographer based in Vermont and Frankfurt), Monica Delgadillo Aguilar (dancer, choreographer, and community dance artist with Tanz die Toleranz, Vienna), Volker Eisenach (community dance choreographer, Berlin), Tamara McLorg (choreographer and community dance artist, Leeds), Patricia Carolin Mai (choreographer and dancer, Hamburg), Royston Maldoom (choreographer and community dance artist, Berlin), and Wilfried van Poppel (dancer, choreographer, and community dance artist, Bremen). All interviews were qualitative and semi-structured expert interviews (Döring and Bortz, 2016) that followed a pre-established interview guide.

The data of this study was primarily collected for a doctoral thesis on the topic of respect behavior. After several steps of feedback within the original research design of the field study, the data on motivation was disclosed. The data was regarded meaningful to the field, in offering insight to the *relevance, inspiration*, and *challenge* for each expert’s perception of motivation to engage in their field. The leading questions during the semi-structured interview addressed the individually perceived reasons to work with heterogeneous groups and/or in the community.

All interviews have been transcribed following the guidelines of Kuckartz (2018) and have been entered into the program for qualitative data analysis MAXQDA. The coding was directed in a cyclical process of qualitative data analysis, following inductive rules of categorizations by Kuckartz (2018) and Rädiker and Kuckartz (2019), while also following Strauss and Corbin (1996) and the methodology of Grounded Theory. To increase the credibility and validity of the analyses, the analyses function “summary grid” has been used and the “code relationship browser,” as well as “statistic for subcodes,” have been applied.

## Results

The results of the qualitative data can be summarized under three major categories regarding the motivation of the experts. Firstly, all seven experts named an *artistic motivation* for choreographing and working with differently abled or heterogeneous groups. Secondly, they all named a *visionary motivation in terms of society.* These included all information on motivation which was driven by the wish and idea of a general frame of social change. Thirdly, from five out of seven interviews, data on *personal or private motivation* for working with this specialized group of people in the field of dance could be collected.

### Artistic Motivation

Focusing on the *artistically* led motivation to work with heterogeneous groups, the expert’s data can be summarized into three sub-categories:

1. *Taking physical differences as creative input*.
2. *To set a focus on creating transformation*.
3. *The participant’s energetic constitutions as enrichment*.

(1) The ability to take all *physical differences* of the participants *as creative input* for choreographing and creating artistic work has been considered by five facilitators as one of the major motivators for dance work in the community. “The love for the challenge to work choreographically with different people” (McLorg, 2019, 39) and the wish to physically engage the participants is what motivates the experts. The aesthetic of a participant’s body who is not afraid of moving is seen as a source for motivation to choreograph, rather than the question of a body’s ability to move: “a body that wants, touches me” (Mai, 2019, 23). Maldoom (2019) even talks about a “high” moment of using these different movement possibilities choreographically:

“It is the challenge of making the best art we can make with people who do not have the same movement language that I have learned. They may not have any movement language that we would associate with contemporary dance or classical dance or whatever. They may not even have any social dance or other kind of dance experience other than maybe, you know, disco dancing or whatever, or freestyle, and that challenge is a bit like a drug” (Maldoom, 2019, 16).

(2) *Setting a focus on creating* an opportunity of *transformation* for the participants has been mentioned by four facilitators as the most important factor of motivation in creating choreographies for heterogeneous groups. The phenomena of transformation, or the “moment of disclosure” (Poppel, 2020, 23) has been defined as the action when participants are suddenly able to follow a movement or instruction at an unusually high level of quality and when they discover their ability to use their own body as an instrument. Usually the participants have not followed these movements or instructions before, due to either emotional or physical conditions which stopped them from transcending their own limits. Overcoming these limits has been acknowledged as a highly motivational factor for the facilitators:

“I also like experiencing the transformation of people I work with (.). I think it is a very, very hard job. But I think at the end, the reward when you see people doing something that they never imagined and resisted imagining that they could do, and doing it at such an extraordinary high level is what makes it so fascinating” (Maldoom, 2019, 16).

The transformation has been set in context with the environment which is created by the experts, which must create space for learning and personal growth for each participant. The focus on an artistic target, rather than a pedagogic mission, has been specifically outsourced as key to initiate that transformation and change:

“For me choreographing within that context within the community context, it is still artistic, because I believe **it is the dance that does the transformation**. It is not me. It is the art and obviously, the art that I work with is in dance. So that is the power. That is the power and I love the challenge. But it is an artistic and choreographic challenge of working with different people (McLorg, 2019, 39).

(3) Finally, *the participant’s energetic constitutions as enrichment* in juxtaposition to the constitutions of professional dancers were named twice as artistic motivational factor for choreographing in the community. “The fascination for the naturalness, which a trained dancer does not bring, because he has already been trained so strongly in his form” (Delgadillo Aguilar, 2019, 21) was mentioned next to the observation that non-professional dancers often share a different, boundless energy when it comes to their first participation in a dance project. This was mentioned as an observable, high energy level, and enthusiasm which lies hidden behind this moment of the “first time” (Eisenach, 2020, 15).

### Visionary Motivation in Terms of Society

The expert’s motivation was–in all seven cases–traceable to an inner wish for change in society. Their motivation was categorized into five sub-categories:

1. *To create new grounds for change (4/7)*.
2. *To change behaviors (2/7)*.
3. *To be inclusive (3/7)*.
4. *To do something which makes sense (2/7)*.
5. *To bring people together (5/7)*.

(1) *Creating grounds for change*, named by four out of seven, was mainly encouraged through the opportunity of working on “new grounds” (Caspersen, 2019, 25) with the participants. The dance space is seen as an environment which is predominantly new to the majority of the groups, so that a lot of enthusiasm and power can be put into stepping into it. Through these new grounds, the facilitators can also encourage the participants to face a challenge or to step into conflict, to confront their own set of behavioral mechanisms or even to cross their own borders. There was an acknowledgment that the dance space, as a ground of change, can be designed or choreographed in a way that it encourages change (ibid.), and that the artform of dance is a predestined ground, which has the opportunity for initiating these kinds of shifts and grounds (McLorg, 2019, 39).

“My interest is primarily in helping other people develop their own ability to step into conflict situations and be able to engage with them productively. To see conflict as a place that can be beneficial, that leads to possibility. Conflict is not failure. Conflict is a natural part of us, learning and changing and growing, it is a part of a creative process” (Caspersen, 2019, 23).

(2) The wish *to change behaviors* has been mentioned twice as another factor for motivation. On the one hand, two facilitators witnessed change of behaviors in the mechanisms of dealing with challenges in the participants in a moment of “how people discover themselves” (Eisenach, 2020, 15) in the dance project. On the other hand, one facilitator also directed the motivation for change of behaviors not only toward the participants but toward himself. He mentioned an opportunity to be constantly open to change one’s own preconditioned perceptions of difference while being the facilitator. Acknowledging the fact that also one’s own perceptions are susceptible to change when working in and with different cultures (Maldoom, 2019, 52) and with heterogeneous groups in general was considered central to the role of being the facilitator:

“(…) when I work in a very different culture: Initially, you will see or feel a difference. But a lot of that difference probably is preconditioned of what you’re expecting. So, you know, you are working with Chinese you have a whole lifetime of building up perceptions of what Chinese are and as they walk in, all those perceptions, glare out at you, the way they look, or the way they behave, or whatever, or in Ethiopia, or if you’re in prison, anywhere. So that can be a first impression of the difference. But what happens, and I’m sure you know this and my other colleagues said this, is that within a couple of days everybody in that project looks like somebody in your last project or project before. So, if you would have a word to describe each person in your last project, you could probably use that same word for at least one person in this project. So instantly you are discovering patterns of human behavior that seem to be absolutely universal” (Maldoom, 2019, 52).

(3) Two experts named a motivation to work with an *inclusive intention*. They are driven by a fundamental belief in participation for all people in arts:

“(…) I totally and utterly believe that everyone should have the opportunity to be involved in the arts.” (McLorg, 2019, 39).

Working inclusively was also defined as a motivation, to work with everyone in the same way, but to bring each and every one differently to the same goal (Mai, 2019, 23).

(4) In two of the interviews, a wish *to do something which makes sense* on a wider social scale while working as facilitator was identified.

“I’m interested in doing it because it makes so much sense. So as an artist I always thought, does it make sense at all, who will look at it? And to do something like that has so much sense and yes, it pleases, it just fulfils me. (…). Of course, I don’t know what it brings for the people, because they have to say that themselves, but for me it brings so much” (Mai, 2019, 23).

The last sub-category which was identified within the category of a visionary motivation in terms of society was named most often. Five out of seven experts refer to be motivated by:

(5) *Bringing people together and* “to bring them to a point, where they can work together for the same goal” (Mai, 2019, 23). These five experts mention a strong belief in encouraging interactions between people with their work. Their data shows that a community dance setting shall be intentionally designed and organized in a way to bring people together and to encourage interaction, reflection, exchange and to further empathy. The increased frequency of interaction has been identified as a supporting motor for understanding and acknowledging the common ground, or universality of individuals (Maldoom, 2019, 52). A fierce belief in the fact that community dance projects are essential for people to interact with each other is mentioned:

“I think it’s imperative that you do that. Because otherwise we fall, many people fall into a parallel society, where you just stay in your own groupings and I think it is not good for a society, if everyone cooks his own soup at home and no exchange takes place. It’s all about exchange. It’s not about convincing someone that the way I live is better. It’s just about promoting some, maybe some kind of empathy or just an understanding for each other. I think it has to be done” (Delgadillo Aguilar, 2019, 27).

### Personal or Private Motivation

Finally, five out of seven experts also mentioned *personally or privately motivated* factors for facilitating dance projects with heterogeneous groups in the community. This was merely defined by a general “interest in people” (Delgadillo Aguilar, 2019, 23), a feeling of “compliance or fulfillment” while doing art that reaches others (ibid.), the previously mentioned motivation for “change of one’s own personal perception of people” (Maldoom, 2019, 52), the positive and exciting feeling toward an “honest and open attitude of the dancers” (van Poppel, 2020, 21), and their own ambitions to *feel, see, or experience* the positive change within the group.

## Discussion

With this research report, more light was shed on the motivation for working with heterogeneous groups and for being the facilitator of the artform dance within heterogeneous groups. According to theory (Atkinson, 1964, p. 1) and the present data, motivation is led by several different factors. The study followed the question of what sort of motivational factors are decisive in leading a dance project with heterogeneous groups and non-professional dancers. Seven interviewed experts shared their visions and personal motivations.

The qualitative data analysis made it possible to extract three key motivational factors for working in highly heterogeneous groups from these seven interviews. These are *artistic motivation, a motivation to initiate change in society, and a personal motivation*. As the artistically based motivation and the motivation to change certain aspects of society were identified in all interviews of the seven facilitators, they can both be regarded as central in order for a facilitator’s motivation to engage in a dance project which addresses the heterogeneous community.

The challenge of working with heterogeneous groups, bodies, and experiences was recognized as a highly positive and fruitful inspiration for choreography and which enriches their work and individual progress as artists. Multiple mentions of transformative moments show that the facilitators are also highly motivated to believe in a great impact–in terms of social and individual change–which their work has on their participants. It can be summarized that the expert’s motivation is guided by a fundamental longing for social change. Looking at the sample, it can even be concluded that it was especially the permanent observation of successful transformation within the group and in single individuals that leads the experts to be so highly motivated to work in their field (Maldoom, 2019, 26). Interestingly, the process of change of behaviors was not defined only as a one-way street directed toward the participants, but has also been followed by a motivation for a change of one’s own behaviors. The process of change was led by an artistic quality which they wish to reveal in their participants, and not through therapeutic or pedagogical techniques.

It can be summarized that success in dance facilitation with heterogeneous groups is always intertwined with the motivation of the facilitator. Future research should now follow up on this connection between the facilitator’s internal personal motivation for change in society, in themselves, and the effect their motivation has on the participants. This could change future discussions not only for inclusion, but also for anti-racism and anti-sexism movement and education. Nevertheless, it has to be noted that the findings of this analysis are predominantly positive regarding the expert’s motivation. It can be assumed that the success and reputation of the chosen artists plays a key role. Further research should therefore collect data from a more diverse group of dance experts, in order to fill this gap. Additionally, it should be acknowledged that this small-scale pilot study can only offer an insight on and from these seven professionals and cannot make claims on behalf of the whole community dance population.

Taking all things into consideration, this research report has underlined that the motivation of the facilitator–especially in cultural education, but presumably for education in general–is extremely relevant for the further development of individuals in groups, and thus also for their future feeling of belonging in our society.

## Data Availability Statement

The raw data supporting the conclusions of this article will be made available by the authors, without undue reservation.

## Ethics Statement

Ethical review and approval was not required for the study on human participants in accordance with the local legislation and institutional requirements. The patients/participants provided their written informed consent to participate in this study. Written informed consent was obtained from the individual(s) for the publication of any potentially identifiable images or data included in this article.

## Author Contributions

The author confirms being the sole contributor of this work and has approved it for publication.

## Conflict of Interest

The author declares that the research was conducted in the absence of any commercial or financial relationships that could be construed as a potential conflict of interest.

## Footnotes

1 The research design of the complete field study included several other data collections from participants during dance projects and were primarily focused on the development of respect during the dance facilitation.
